# The Vulnerability of the Developing Brain: Analysis of Highly Expressed Genes in Infant C57BL/6 Mouse Hippocampus in Relation to Phenotypic Annotation Derived From Mutational Studies

**DOI:** 10.1177/11779322211062722

**Published:** 2022-01-05

**Authors:** Angelica Lindlöf

**Affiliations:** School of Bioscience, University of Skövde, Skövde, Sweden

**Keywords:** Hippocampus, postnatal development, infant, phenotypic annotation, mutational studies, mouse

## Abstract

The hippocampus has been shown to have a major role in learning and memory, but also to participate in the regulation of emotions. However, its specific role(s) in memory is still unclear. Hippocampal damage or dysfunction mainly results in memory issues, especially in the declarative memory but, in animal studies, has also shown to lead to hyperactivity and difficulty in inhibiting responses previously taught. The brain structure is affected in neuropathological disorders, such as Alzheimer’s, epilepsy, and schizophrenia, and also by depression and stress. The hippocampus structure is far from mature at birth and undergoes substantial development throughout infant and juvenile life. The aim of this study was to survey genes highly expressed throughout the postnatal period in mouse hippocampus and which have also been linked to an abnormal phenotype through mutational studies to achieve a greater understanding about hippocampal functions during postnatal development. Publicly available gene expression data from C57BL/6 mouse hippocampus was analyzed; from a total of 5 time points (at postnatal day 1, 10, 15, 21, and 30), 547 genes highly expressed in all of these time points were selected for analysis. Highly expressed genes are considered to be of potential biological importance and appear to be multifunctional, and hence any dysfunction in such a gene will most likely have a large impact on the development of abilities during the postnatal and juvenile period. Phenotypic annotation data downloaded from Mouse Genomic Informatics database were analyzed for these genes, and the results showed that many of them are important for proper embryo development and infant survival, proper growth, and increase in body size, as well as for voluntary movement functions, motor coordination, and balance. The results also indicated an association with seizures that have primarily been characterized by uncontrolled motor activity and the development of proper grooming abilities. The complete list of genes and their phenotypic annotation data have been compiled in a file for easy access.

## Introduction

The hippocampus has been shown to be important for learning, long-term memory formation and spatial navigation. Moreover, being a part of the limbic system, it has also been shown to participate in the regulation of emotions, by attaching experienced emotions and senses to the memories.^1-3^ However, its specific role(s) in memory is still unclear.^4^ Hippocampus is physically connected to other brain structures,^5^ for example, the neocortex^6^—important for sensory perception, spatial reasoning, and language; the amygdala^7^—which regulates emotional behavior; and the thalamus—responsible for relaying motor and sensory signals to cerebral cortex, but is also involved in the regulation of sleep, alertness, and arousal mechanisms.^8^ Through these connections, the brain structures stimulate each other in an intricate manner to execute cognitive processes.^1,2,9-17^ A damage or dysfunction in one structure can heavily affect the function of another.

Hippocampal damage or dysfunction mainly results in memory issues, especially in the declarative memory, and in severe cases amnesia, but has in animal studies also shown to lead to hyperactivity and difficulty in inhibiting responses previously taught.^18-27^ The brain structure is affected in neuropathological disorders such as Alzheimer’s, epilepsy, and schizophrenia but also by depression and stress.^12,14,25,27-39^

The hippocampus structure is far from mature at birth and undergoes substantial development throughout infant and juvenile life, with gross morphological changes.^40-47^ The major neurogenesis of the structure has, however, been established to occur prenatally; although there is evidence that vital production of new neurons also occurs postnatal.^48^ More importantly, abnormalities in postnatal development of the hippocampus are thought to contribute to neurodevelopmental disorders, such as autism.^49-52^

The aim of this study was to survey genes highly expressed throughout the postnatal period in hippocampus and which have also been linked to an abnormal phenotype through mutational studies, to achieve a greater understanding about hippocampal functions during postnatal development. In this study, previously produced gene expression data from *C57BL/6 mouse hippocampus* was used and analyzed.

Mouse is one of the most commonly used models for human biology, and the C57BL/6 mouse strain is one of the most widely used inbred strain for genetic studies.^53,54^ This strain is the preferred choice as background for genetically modified mice, due to its availability of congenic strains, easiness to breed, being genetically stable, and having a low susceptibility to developing tumors. However, the strain has also less-attractive characteristics, such as a high susceptibility to diet-induced obesity and diabetes, sensitivity to pain and loud noise, having a tendency to bite, and susceptibility to morphine addiction. Nonetheless, the strain has been appreciated for behavioral studies, as it is physically active, a good learner, has a relatively high level of social exploration and is relatively stress-resistant. Due to its popularity, there is a substantial amount of gene expression data available for the *C57BL/6* strain and which are also easy to access.

Commonly when conducting genome-wide gene expression studies, differentially expressed genes between different conditions are identified and analyzed. However, in this study, another approach was chosen, by focusing on the most highly expressed genes throughout the postnatal period and the phenotypes that can be linked to these genes. Highly expressed genes are considered to be of potential biological importance and appear to be multifunctional, and hence, any dysfunction in such a gene will most likely have a large impact on the development of abilities during the postnatal and juvenile period.^55-63^ Phenotypes derived from mutational studies of these genes can contribute to more knowledge regarding the functions of hippocampus.

The study was limited to gene expression data derived using the Affymetrix GeneChip Mouse Genome 430 2.0, as an attempt to avoid deviations between different platforms as well as arrays having a smaller number of represented genes.

In total, 5 time points, at postnatal day (PD) 1, 10, 15, 21 and 30, covering 15 samples from 3 different studies were selected to be included in the analyses.^64-66^ The most highly expressed genes in all of these time points were derived and analyzed. Phenotypic annotation data were derived from Mouse Genomic Informatics (MGI) database and limited to include only hetero- or homozygous/wild-type gene mutational studies.^67^

## Materials and Methods

### Gene expression data

Gene expression data were downloaded from ArrayExpress^68,69^ using the following criteria: mouse strain C57BL/6 or any substrain, Affymetrix GeneChip Mouse Genome 430 2.0, hippocampus tissue, time point(s) ⩽30 PDs and at least 2 biological replicates. A quality control of each microarray/sample was performed using *R* and the package *simpleaffy*.^70^ Microarrays meeting the following criteria were excluded from the analyses: background quality >90, beta actin ratio >**|**3**|** and GADPH ratio >**|**1|. This resulted in the following experiments to be included in the analyses: E-GEOD-21137 (PD ~21; 2 biological replicates), E-GEOD-49050 (PD 1, 15, and 30; 3 biological replicates per time point) and E-GEOD-48911 (PD 10; 4 biological replicates).

Gene expression values were derived for all probes using *R* and *simpleaffy*,^70^ with robust multiarray average (RMA) as normalization method. Microarrays for each experiment with chosen time points were normalized separately.

### Highly expressed genes

For each time point and biological replicate/sample, the 1500 most highly expressed probes were obtained, by ranking the probes according to derived normalized expression value. Thereafter, only probes being among the most highly expressed in all samples, except for the 3 samples from PD 1, were included in further analyses. From this subset, probes not annotated as protein coding in the MGI database^67^ were excluded. More specifically, a list of the probes’ Affymetrix microarray IDs was submitted to MGI’s Batch Query tool (MGI Batch Query [jax.org]), to retrieve gene annotation data; those probes not annotated with the Feature Type term “protein coding gene” were removed. From this batch query, MGI Gene/Marker ID for each probe was also extracted. Thereafter, redundant probes referring to the same gene name and MGI ID were removed, so that only one unique gene name/MGI ID was kept for subsequent analyses. The final set of genes was termed Unique Highly Expressed Genes (UHEGs).

### Gene annotation data

Phenotypic annotation data were downloaded from MGI’s webpage, using the MGI Data and Statistical reports page (MGI Data and Statistical Reports [jax.org]) and the file “List of all mouse phenotypic alleles.” From this list, only annotation derived from hetero- or homozygous/wild-type mutational studies were included. To note, these studies include other strains in addition to *C57BL6*. Subsequently, from this list, only annotations for the UHEGs were extracted in the form of Mammalian Phenotype IDs.

The Mammalian Phenotype ID refers to a term in the hierarchically structured Mammalian Phenotype Ontology (MPO)^71^ and is used for annotating genes. In this study, all terms for all genes were downloaded as well as each term’s parental terms. Redundant terms were removed for each gene, and the terms “normal phenotype” and “no phenotypic analysis” as well as their child terms were excluded from subsequent analyses. Subsequently, terms down to child level 4 in the hierarchical structure were included in the study.

### Housekeeping genes

List of housekeeping genes in mouse was downloaded from the HRT Atlas v1.0 database,^72^ and their respective gene names were searched for in the current NetAffx annotation file for Affymetrix GeneChip Mouse Genome 430 2.0 Array (http://www.affymetrix.com/support/technical/byproduct.affx?product = moe430-20). Probe IDs for the housekeeping genes that could be identified in the annotation file was extracted, and their normalized (unintegrated) expression values were derived. The extracted probe IDs were subsequently used for identifying which ones of them referred to a UHEG.

### Statistical data analysis and visualization

Violin plots were generated with BoxPlotR, a web tool for generation of box plots (http://shiny.chemgrid.org/boxplotr/).^73^ Pairwise annotation term combinations were produced using an in-house developed Perl script. Gene networks were visualized with Cytoscape using the Edge-weighted spring-embedded layout with weights as parameter.^74^

## Results

### Mouse postnatal hippocampus microarray data

In this study, publicly available microarray gene expression data from mouse postnatal hippocampus was used as basis; a search in ArrayExpress using the criteria “*C57BL6; Affymetrix GeneChip Mouse Genome 430 2.0; hippocampus*” resulted in 35 available experiments. However, 31 of these experiments did not include tissue samples from PDs and were, consequently, excluded from the study. The remaining 4 experiments were subjected to a quality control of the microarray data, using the package *simpleaffy* in *R*.^70^ Only time points ⩽30 PDs and from normal hippocampus tissue were included in the study, and each experiment with selected time points were controlled separately. The quality control showed that data from one of the 4 experiments was poor, as it had a *background quality* >90, *beta actin ratio* >**|**3**|** and *GADPH ratio* >**|**1**.|** Hence, this data set was excluded from subsequent analyses. The remaining 3 experiments included 5 time points (1, 10, 15, 21 and 30 PDs) and with 2-4 biological replicates each, yielding in total 15 samples (Table 1).

**Table 1.** Genome-wide gene expression experiments evaluated for subsequent gene expression analyses.

Experiment	Strain	Assays		Age	Quality		
		Total	Anal.		BG	β-actin	GADPH
E-GEOD-21137	C57BL/6 J	16	2	3 weeks	0	0	0
E-GEOD-48911	C57BL/6	31	4	10 days	0	0	0
E-GEOD-49050	C57BL/6	72	9	1, 15, 30 days	0	0	0
E-GEOD-61086	C57BL/6	14	3	1 weeks	0	3	3

Experiment, refers to experiment’s ArrayExpress ID; Strain, mouse strain used in experiment; Assays Total, total number of samples/individuals used in original experiment; Assays Anal., number of samples/individuals selected for this study (according to appropriate time point); Age, age of the samples/individuals that were selected for this study; Quality, number of samples/individuals with a bad quality values, background **>** 90, β-actin **>**|3| and GADPH **>**|1.|

To extract the most highly expressed genes, first, probe expression values were derived using the *simpleaffy* package in *R* and normalized with the robust multi-array average RMA method (each data set was normalized separately).^70^ Thereafter, probes being among the top 1500 most highly expressed in all replicates for time points 10, 15, 21 and 30 PDs were derived. From this set, only those probes referring to a protein coding gene in the MGI database were included in subsequent analyses, that is, all probes having an MGI Feature Type annotation “protein coding gene.”^67^ Some of these probes referred to the same gene and subsequently redundant probes were removed, so that the final set comprised of only unique genes. This final set included 547 genes and is the basis of the subsequent annotation analyses. This gene set was termed UHEGs (Supplementary File 2).

### UHEGs annotated with an abnormal phenotype

To identify if any of the UHEGs had previously been associated with an abnormal phenotype, annotation was extracted from the MGI’s Mammalian Phenotype database.^67,71^ The MPO is a hierarchically structured vocabulary used for standardized annotation of mouse genotypes. Genes (alleles) are annotated with high-level broadly descriptive phenotypic terms down to low-level highly specific ones and where the lower-level more detailed term is a child of a more general descriptive term. In this study, only annotation derived from hetero- or homozygous/wild-type mutational studies was included and not, for example, more complex studies involving more than one gene or allele. The phenotype data include studies on other strains in addition to *C57BL6*.

For the UHEGs, all MPO terms were extracted for each gene, as well as all parental terms to these ones. Thereafter, terms down to child level 3 (in total 4 levels including top level terms) in the hierarchical structure were included in subsequent analyses, to limit the amount of data to be analyzed. Level 4 in the hierarchical tree of the MPO will give sufficiently detailed annotation. Since the ontology is based on a hierarchical structure and a gene could have been associated with more than one phenotype, this procedure results in redundancy and therefore, subsequently, all redundant terms were removed for each gene. In addition, the terms “normal phenotype” and “no phenotypic analysis” as well as their child terms were excluded from the analyses. In total, for 348 (63%) of the 547 UHEGs at least one mutant had been generated and annotated, and moreover, 283 (52%) of the UHEGs had been associated with at least one abnormal phenotype (ie, after excluding the terms “normal phenotype” and “no phenotypic analysis”). The list of UHEGs with their abnormal phenotype annotations and normalized (unintegrated) expression levels has been compiled for easy access and can be found in Supplementary File 4.

### Phenotype annotation statistics

Most of the UHEGs (57%) with a phenotype annotation have been associated with 1 to 5 terms and generally reported in 1 to 2 mutational studies (Supplementary File 1). Only 7% (39 genes) have been reported for more than 5 mutational studies and 28% (155 genes) in only one study. On the contrary, there are a few genes that have been associated with a very large number of abnormal phenotypes. The most studied genes are *Apoe, Prnp*, and *Thra*, which have been included in 82, 24, and 23 different mutational studies, respectively. However, the genes with the greatest number of reported abnormal phenotypes are *Ctnnb1, Rpl38*, and *Thra*, with 40, 32, and 27 different terms, respectively, on child level 4 in the MPO. Regarding *Apoe, Prnp*, and *Thra*, these genes have been annotated with a slightly less number of child-level 4 terms: 24, 14, and 27 different phenotypes, respectively.

The distribution of the number of annotated terms per gene follows the classical hypergeometric for genomic data, that is, most of the genes have been annotated with a few terms and a small number of genes have been annotated with a very large number of terms (Supplementary File 1). Here, it clearly reflects a bias in the number of mutational studies carried out for a gene and hence, the knowledge that exists for each gene.

### Housekeeping genes

Since the genes selected were highly expressed in all included time points, they were also stably expressed; in total, 97% of the genes have a normalized (unintegrated) expression variance <1. Genes that maintain constant expression levels across many different conditions could represent housekeeping genes. To investigate the presence of such genes, the UHEGs were compared to suggested housekeeping genes in the HRT Atlas v1.0 database (Supplementary File 5).^72^

Of the 3024 housekeeping genes listed in HRT Atlas v1.0,^72^ only 31 could not be identified in the annotation file for the Affymetrix GeneChip Mouse Genome 430 2.0. For the remaining housekeeping genes, these referred to 6659 probes, and hence, some of them are represented by more than one probe on the chip. Regarding the probes, 96% of them have a normalized (unintegrated) expression variance <1, showing that most of the suggested housekeeping genes have a stable expression across the samples analyzed in this study.

Regarding UHEGs, in total, 131 (24%) of them are represented in the list of suggested housekeeping genes from the HRT Atlas v1.0.^72^ Hence, most of the suggested housekeeping genes are not highly expressed in the analyzed samples and most of the derived UHEGs are not listed as a housekeeping gene in the database. Furthermore, 29 of these genes have no annotated abnormal phenotype, resulting in 102 (19%) UHEGs as plausible housekeeping genes with at least one annotated abnormal phenotype.

### Phenotype annotation analyses

Analyses of the MPO annotation reveal that all 27 first-level terms in the hierarchical structure has at least one gene associated to it; however, some terms have more genes associated than others. The most common abnormal phenotype for the UHEGs, based on number of genes annotated with the term, is “mortality/aging,” followed by “nervous system,” “growth/size/body region” and “behavior/neurological” (Table 2). Regarding second-level terms, the most common phenotypes are “abnormal survival,” “abnormal behavior,” and “abnormal nervous system,” and for third-level terms these are “abnormal motor capabilities/coordination/movement,” “abnormal brain morphology,” and “abnormal neuron morphology” (Supplementary File 2).

**Table 2.** Phenotype annotation derived from the MGI database for the unique highly expressed genes (UHEG) for the first-level terms.

MPO term	# genes	% genes
Mortality/aging	151	28%
Nervous system phenotype	136	25%
Behavior/neurological phenotype	123	22%
Growth/size/body region phenotype	102	19%
Cellular phenotype	97	18%
Homeostasis/metabolism phenotype	80	15%
Embryo phenotype	58	11%
Cardiovascular system phenotype	57	10%
Muscle phenotype	50	9%
Reproductive system phenotype	43	8%
Hematopoietic system phenotype	41	7%
Immune system phenotype	37	7%
Vision/eye phenotype	28	5%
Liver/biliary system phenotype	27	5%
Respiratory system phenotype	27	5%
Adipose tissue phenotype	25	5%
Skeleton phenotype	25	5%
Integument phenotype	20	4%
Craniofacial phenotype	19	3%
Digestive/alimentary phenotype	18	3%
Endocrine/exocrine gland phenotype	18	3%
Renal/urinary system phenotype	18	3%
Limbs/digits/tail phenotype	15	3%
Hearing/vestibular/ear phenotype	14	3%
Neoplasm	13	2%
Pigmentation phenotype	13	2%
Taste/olfaction phenotype	9	2%

Abbreviations: MGI, Mouse Genomic Informatics; MPO, mammalian phenotype ontology; UHEG, unique highly expressed genes.

MPO term, phenotype term used in MGI; MPO ID, phenotype term ID used in MGI; # UHEG, number of UHEG annotated with the term; % UHEG, percentage number of UHEG annotated with the term; MPO level, child level in the MGI ontology hierarchy.

There are in total 473 4-level terms with at least one UHEG associated to it, but only 269 (49%) of the UHEGs have been annotated with a 4-level term. Here, “abnormal voluntary movement” is the most occurring as 64 of the UHEGs (12%) have been annotated with the term. This term is followed by “abnormal forebrain morphology” (48 UHEGs, 9%) and “abnormal motor coordination/balance (44 UHEGs, 8%) (Supplementary File 2). Moreover, on this level, the fourth and fifth most occurring terms are “preweaning lethality, complete penetrance” (42 UHEGs, 8%) and “abnormal learning/memory/conditioning” (38 genes, 7%).

The union of all genes annotated with the top-5 most occurring 4-level terms comprises 139 (26%) of the UHEGs. However, none of these genes have been annotated with all 5 terms, and only 11 of them have been annotated with at least 4 terms and 25 with at least 3 terms.

### Relationship between MPO terms

On the contrary, lists with most annotated terms do not capture the relationships that exist between terms on different levels, which in turn do not reflect the complexity of hippocampus’ functional roles. To meet this limitation, the hierarchical tree of MPO terms was pictured together with the relationships between terms down to child-level 4; a node in the tree represents an abnormal phenotype annotation and a directed edge represents the relation between a parent and child term, that is, the edge represents the *is_a* relation from the MPO. In addition, the size of a node represents the number of UHEGs that have been annotated with that term. In total, 27 such hierarchical trees were generated, one for each of the first-level terms and their respective child levels. However, as it is not possible to include all these trees here, only some of them will be highlighted and analyzed.

**Figure 1.**
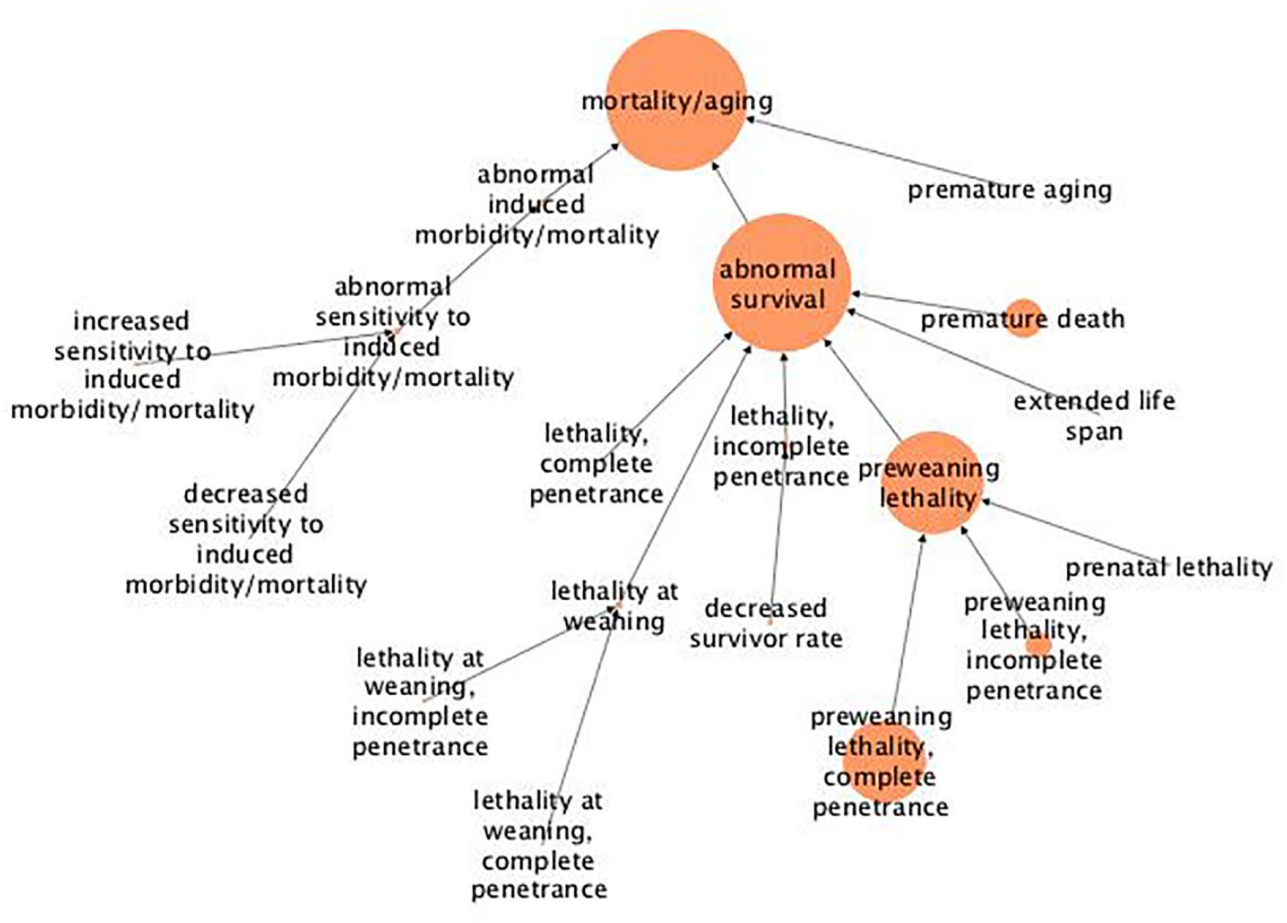
Hierarchical tree for the abnormal phenotype “mortality/aging.” The figure shows results from abnormal phenotype annotation of the UHEGs depicted with the MPO relations and where a larger circle indicates more genes being annotated with the specified term. MPO indicates mammalian phenotype ontology; UHEG, unique highly expressed genes.

Regarding “mortality/aging,” the most occurring term, the mutational effects mainly refers to lethality prior to or shortly after birth, as most of the genes have been annotated with terms related to preweaning lethality and premature death, and not with other terms related to a later death, such as at or after weaning age (Figure 1).

**Figure 2.**
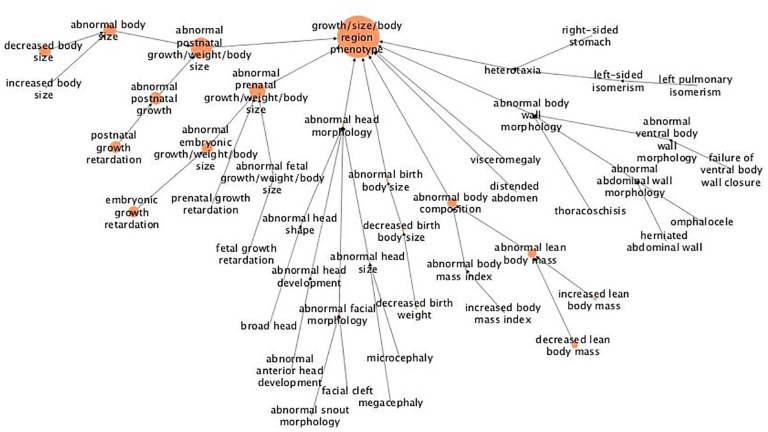
Hierarchical tree for the abnormal phenotype “growth/size/body region.” The figure shows results from abnormal phenotype annotation of the UHEGs depicted with the MPO relations and where a larger circle indicates more genes being annotated with the specified term. MPO indicates mammalian phenotype ontology; UHEG, unique highly expressed genes.

Regarding “growth/size/body region,” it can be seen that the gene mutations affect growth and development of body size, both prior (prenatal) and after birth (postnatal) (Figure 2). Moreover, the mutations mainly seem to result in a growth retardation, rather than an increase of body size, since most of these alleles have been annotated with terms related to retardation during different development phases, including embryonic, prenatal, and postnatal. There is also an association to a decreased lean body mass.

**Figure 3.**
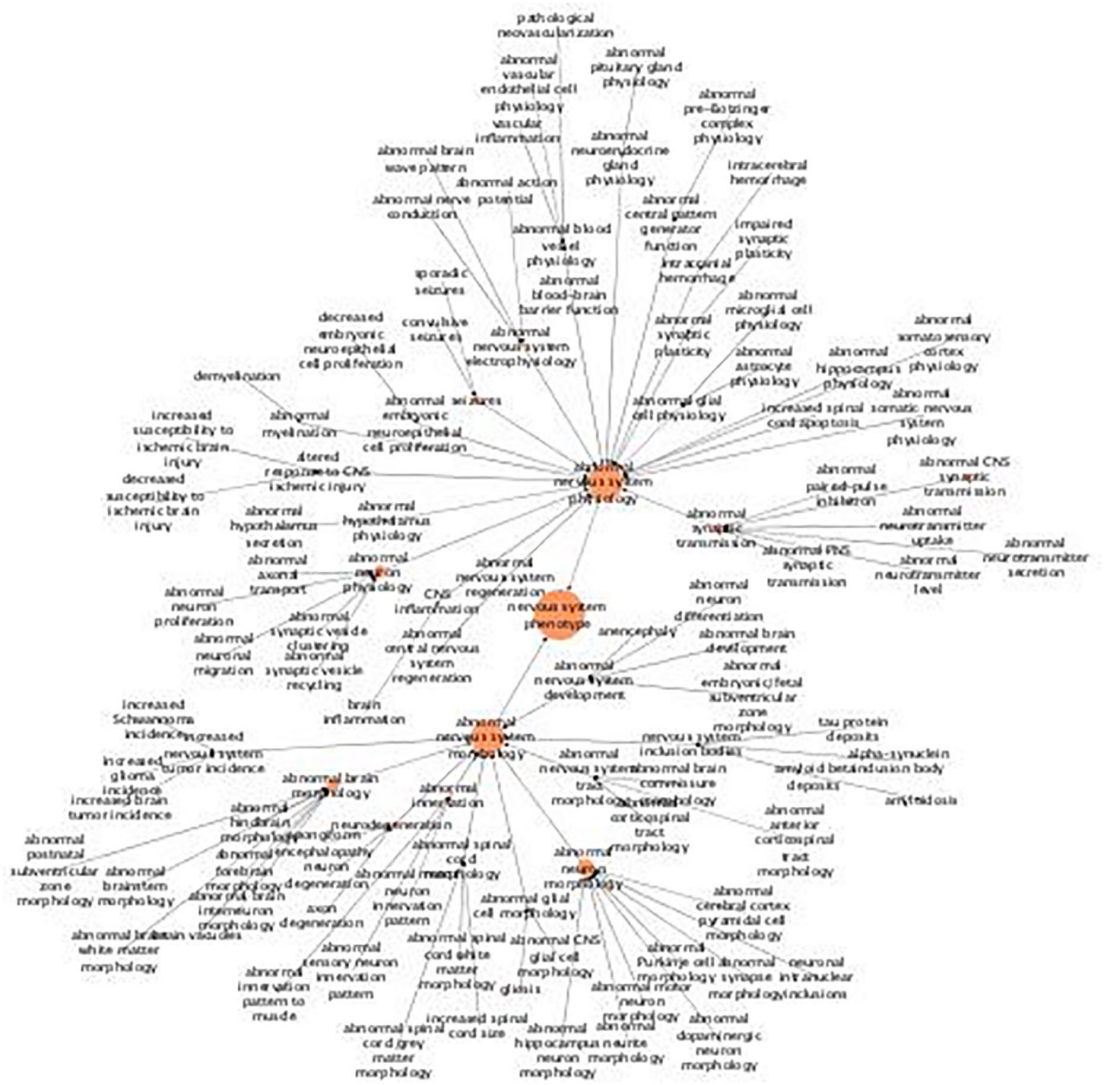
Hierarchical tree for the abnormal phenotype “nervous system.” The figure shows results from abnormal phenotype annotation of the UHEGs depicted with the MPO relations and where a larger circle indicates more genes being annotated with the specified term. MPO indicates mammalian phenotype ontology; UHEG, unique highly expressed genes.

For “nervous system” phenotype, there are substantially more terms included from the MPO (Figure 3). Hence, this tree is larger than the 2 previous ones with respect to the number of nodes. On the contrary, each term has a smaller number of genes associated to it. In a sense, this makes it more difficult to derive a cohesive picture of the mutational effects. Or, the other way around, it shows that these alleles have a variety of functional roles in the nervous system. But nonetheless, there are some commonalities that can be derived from the tree. There are a number of alleles that affect both the morphological and physiological development of different brain structures, hippocampus included, which indicates that these UHEGs are important for the development of other structures besides hippocampus. There is an apparent association with hippocampus and the spinal cord/central nervous system because several of the genes have been annotated with abnormal phenotypes related to these structures. There are some alleles which affect synaptic morphology, transmission, plasticity, and/or vesicle transport as well as those affecting the formation of myelin sheaths and/or insulation. There is also an association with hippocampus and voluntary movements, via alleles giving rise to abnormalities in motor neuron morphology, and/or the corticospinal tract as well as the somatic nervous systems. To this can also alleles which give rise to seizures be grouped because these seizures have primarily been associated to those characterized by uncontrolled motor activity.

**Figure 4.**
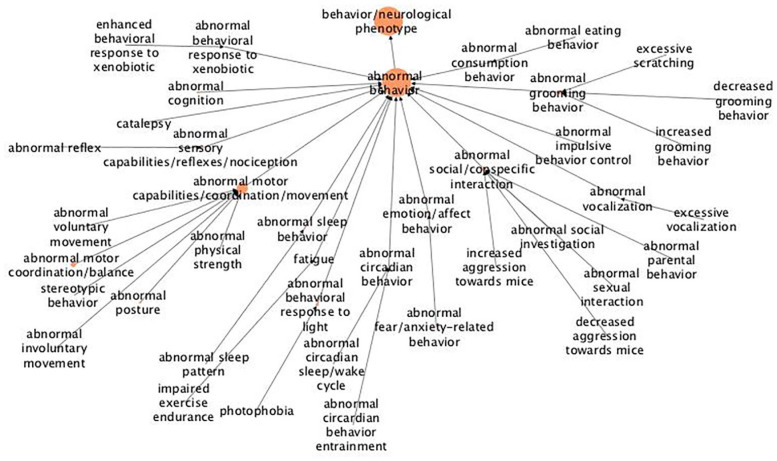
Hierarchical tree for the abnormal phenotype “behavior/neurological.” The figure shows results from abnormal phenotype annotation of the UHEGs depicted with the MPO relations and where a larger circle indicates more genes being annotated with the specified term. MPO indicates mammalian phenotype ontology; UHEG, unique highly expressed genes.

For “behavior/neurological phenotype,” the tree can actually be divided into several subgroups based on a common theme (Figure 4). There is one group of genes that are linked to the control of bodily movements, including coordination of voluntary and involuntary movements, balance and posture, reflexes and catalepsy. Another theme comprises genes linked to emotions and social interactions, more specifically fear and anxiety, aggression and social investigation. A third group of genes are linked to behavior control and which mainly comprises the control of grooming, social/con-specific interactions, and consumption. There is one theme related to fatigue and sleep/wake cycle, which may also be grouped together with behavior related to the circadian rhythm and response to light.

**Figure 5.**
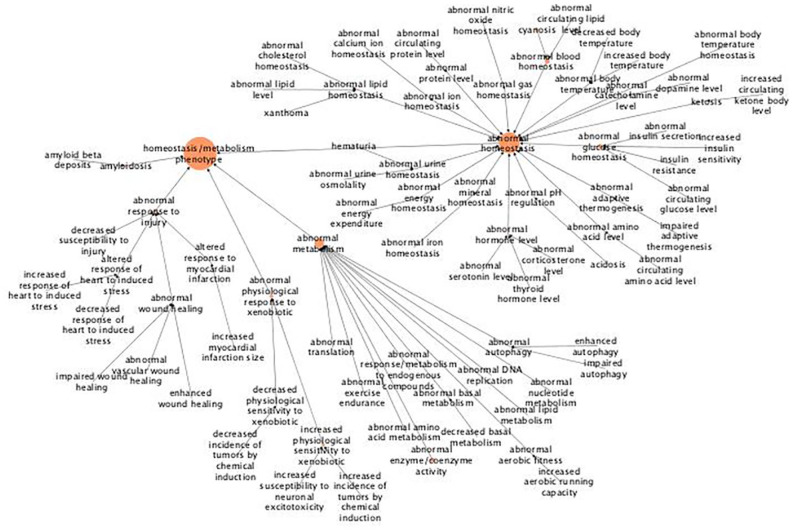
Hierarchical tree for the abnormal phenotype “homeostasis/metabolism.” The figure shows results from abnormal phenotype annotation of the UHEGs depicted with the MPO relations and where a larger circle indicates more genes being annotated with the specified term. MPO indicates Mammalian Phenotype Ontology; UHEG, Unique Highly Expressed Genes.

Similar to “nervous system,” the “homeostasis/metabolism” MPO includes more terms, but with fewer genes annotated for each term (Figure 5). For this MPO, the genes have mainly been annotated with “abnormal homeostasis” and “abnormal metabolism,” although there are a few genes linked to responses to physical injury as well as to xenobiotics and maintenance of body temperature. Regarding “abnormal homeostasis,” most of these genes have been associated with maintenance of blood composition (“abnormal blood homeostasis”), which in turn mainly seem to involve oxygenation of the blood (“cyanosis”), with maintenance of glucose levels (“abnormal glucose levels”), which in turn mainly seem to be related to proper insulin function, and to some extent maintenance of hormone levels, which includes serotonin, thyroid, corticosterone, and dopamine.

## Discussion

The aim of this study was to gain a greater understanding about hippocampal functions during postnatal development, by surveying protein coding genes highly expressed throughout this time period in mouse and use annotation about abnormal phenotypes that had been obtained through mutational studies. Mouse is one of the most commonly used models for human physiology and disease, and especially the C57BL/6 is the one most widely used inbred strain for genetic studies.^53,54^ Moreover, the MGI database contains a wealth of data from various experimental studies and contains a comprehensive catalog of mutant alleles, which are also associated to their phenotype through the MPO.^67,71^ Due to the strain’s popularity, there is also a substantial amount of gene expression data available that can be easily accessed.^68,69^

Previous gene expression studies on postnatal development in mouse hippocampus have mainly focused on the response to a specific exposure, for example, ethanol, iron, and nicotine exposure,^75-79^ or a specific gene expressed during postnatal development and/or in the context of a specific neurological disease or disorder.^25,62,65,66,80-85^ The study by Mody et al^86^ focused on genes differentially expressed between different time points during postnatal development (<30 PD) in mouse hippocampus, but did not include any analysis of genes highly expressed over several time points nor relating the results to phenotypic data. Similarly, the study by Iacono et al^87^ focused on genes differentially expressed during different PDs in mouse hippocampus, with the added dimension of studying single-cell RNA-seq data from various hippocampal cell types but did not include any analysis of highly expressed genes nor phenotypic data. The work by Meyer^57^ focused on genes highly expressed within the hippocampal sector CA1 identified from a mouse brain histological expression atlas including 1013 genes; in total, 16 genes were found to be highly expressed at both 7 PD and in adult mice. However, this study was somewhat limiting as it only included a smaller number of genes and only one time point from the postnatal period, nor did the study include any phenotypic data. Contrarily, the study presented here focus on the analysis of phenotype annotations derived from hetero- or homozygous/wild-type mutational studies for 547 genes found to be highly expressed during the first 30 PD, adding new valuable information about the roles of hippocampus.

On the contrary, this study was limited to protein coding genes and future work should include similar analyses on noncoding RNA (ncRNA) expression, such as microRNAs and long noncoding RNAs, as previous studies have shown that such RNAs are expressed in a tissue- and cell-specific manner, involved in neuronal differentiation and function, and implicated in various brain disorders.^88-95^ However, although databases providing information on ncRNAs along with tissue expression profiles and predicted functions are now available as well as ncRNA knockout mouse models with reported phenotypes,^96-101^ there is still a lack of databases that compiles the results from mutational studies and which also links the ncRNAs to phenotype in a similar way as the MGI’s Mammalian Phenotype database. Such databases would be of high value when further studying the roles of hippocampus.

The analysis of the derived phenotype data for the UHEGs revealed that for 63% of them, a mutant had been generated (including those where no abnormal phenotype was detected), and in total, 52% of the UHEGs had been associated with an abnormal phenotype (excluding those where no abnormal phenotype was detected). Moreover, most of these genes had been reported in 1 to 2 mutational studies, and there were only a minor portion of the UHEGs for which there were more than 5 mutational studies. Clearly, there are ethical issues to consider when subjecting animals to experimental studies and such studies should always only be carried out when there is a possibility to generate data that will be useful for a treatment in humans.^102^ However, for some of the UHEGs, a very large number of mutational studies have been conducted (14 of the UHEGs in at least 10 different studies), and there is also a minor portion of the UHEGs that have been annotated with a very large number of abnormal phenotypes (only 26 of the UHEGs have at least 10 4-level terms). Such an apparent bias toward a minor portion of the genes under study obscures an objective analysis of the data and limits in-depth analyses.^103^ It is difficult to get a broad and detailed understanding for all of these genes’ phenotypic roles because there is only annotation available for about half of the genes and the amount of annotation deviates to such a large extent among these genes; there is a true challenge in analyzing genetic data.

Since the UHEGs showed a stable expression across all time points analyzed, these could be housekeeping genes, which are genes defined as constitutively expressed across different conditions and tissues and mainly required for the maintenance of basic cellular functions. As such they could introduce a bias in the results toward genes that are highly expressed in hippocampus regardless of developmental stage, rather than being involved in the development of hippocampal functions during postnatal period. A portion of the UHEGs (24%) are plausible housekeeping as they are listed in the HRT Atlas v1.0 database.^72^ However, some of these genes did not have any annotated abnormal phenotype in the MGI’s Mammalian Phenotype database, leaving 19% of the UHEGs as plausible housekeeping with a resulting abnormality. Furthermore, most of the suggested housekeeping genes listed in the HRT Atlas v1.0 database do not seem to be highly expressed during the first 30 PD in hippocampus, at least based on the samples included in this study. On the contrary, the concept “housekeeping gene,” as Hounkpe et al^72^ pointed out, appears to be problematic as the concordance between previous studies aimed at identifying such genes is low and the number of false positives is a major issue. Interestingly, Hounkpe et al^72^ introduced a redefined definition of the concept “housekeeping genes” and identified a number of such genes based on this new definition, which are stored in the HRT Atlas v1.0 database and on which the identification of plausible housekeeping genes in this study is based on. In addition, previous studies have shown that housekeeping genes can be involved in multicellular organogenesis, neurogenesis, developmental processes, and affected by disease.^104-113^ Therefore, the identified plausible housekeeping genes among the UHEGs were not automatically ruled out as being involved in the development of hippocampal functions during the postnatal period.

Regarding the abnormal phenotype analyses, there are several interesting results that can be summarized from this study and which will be outlined here. Many of the genes highly expressed during the postnatal period give rise to similar abnormal phenotypes when mutated and have a great overall effect on the individual. As previously described highly expressed genes appear to be multifunctional and that is also shown in the results here.^55-63^ A mutation in as many as 151 of these genes can lead to a fatal outcome primarily before or shortly after birth. Hence, the abnormal phenotype of their alleles suggests that they are involved in proper embryo development and infant survival. Interestingly, previous studies have associated hippocampus malformation with sudden death in infants (sudden infant death syndrome, SIDS) and early childhood (sudden unexplained death in childhood, SUDC), and where the 2 syndromes demonstrate similar hippocampal abnormalities.^81,114,115^

The results for the abnormal phenotype “growth/size/body region” indicates that many of the highly expressed genes are involved in proper growth and increase in body size, since mutations in these genes mainly led to growth retardation, decrease in body size and lean body mass. Previous studies have shown that fetal growth restriction (also known as intrauterine growth restriction) affects the development of hippocampus and is associated with a decreased hippocampus volume but is also associated with an overall reduced brain volume, abnormalities in the development of white matter myelination and the basal ganglia.^47,116-122^ Moreover, fetal growth restriction has been shown to lead to a reduction in motor capabilities, cognition, and learning as well as behavioral issues such as poor attention and altered mood.^116-123^

Interestingly, the results indicate a plausible association between hippocampus and the spinal cord. There are also some mutant alleles that gave rise to abnormalities in motor neuron morphology, corticospinal tract, and somatic nervous system. All these parts are vital for proper functioning of voluntary movements, body/motor coordination, and balance.^124-130^ There were also some alleles which gave rise to seizures, and to note, such seizures that primarily have been characterized by uncontrolled motor activity.^33,131,132^ In addition, the results showed that some of the mutant alleles gave rise to behavioral abnormalities related to motor capabilities, coordination, movement, and balance (see results for abnormal phenotype “behavior/neurological”). Previous studies have shown that hippocampus is involved in motor sequence memory consolidation, voluntary movements, motor balance and coordination, and that there is an association between epilepsy and hippocampus.^33,35-37,133-142^ The results here also indicate a connection between hippocampus, voluntary movements, body coordination, and balance, as well as between epilepsy which causes motor seizures, but this has to be further studied before any conclusions can be made.

Another interesting aspect drawn from the results is that a portion of these genes seems to be important for developing grooming abilities, which is a behavior that functions to maintain hygiene, comfort, and social communication. Grooming can also be considered a physical activity where repeated stereotyped movements are executed as a complex sequenced structure.^143^ The execution of these movements requires an intricate pattern of motor activities and motor control. Previous studies have shown that damages in and degeneration of hippocampus lead to alterations in grooming activities, such as fewer, shorter, and uncomplete grooming sequences.^144-147^ The results here indicate a connection between genes expressed in hippocampus and proper development of grooming abilities, but this also has to be further studied before any conclusions can be made.

## Conclusions

This study suggest that genes highly expressed in mouse hippocampus during postnatal period are important for proper embryo development and infant survival, growth, and increase in body size, as well as for voluntary movement functions, motor coordination, and balance. The results also indicated an association with seizures that have primarily been characterized by uncontrolled motor activity and the development of proper grooming abilities.

## Supplemental Material

sj-jpg-1-bbi-10.1177_11779322211062722 – Supplemental material for The Vulnerability of the Developing Brain: Analysis of Highly Expressed Genes in Infant C57BL/6 Mouse Hippocampus in Relation to Phenotypic Annotation Derived From Mutational Studies Click here for additional data file. Supplemental material, sj-jpg-1-bbi-10.1177_11779322211062722 for The Vulnerability of the Developing Brain: Analysis of Highly Expressed Genes in Infant C57BL/6 Mouse Hippocampus in Relation to Phenotypic Annotation Derived From Mutational Studies by Angelica Lindlöf in Bioinformatics and Biology Insights

sj-xlsx-2-bbi-10.1177_11779322211062722 – Supplemental material for The Vulnerability of the Developing Brain: Analysis of Highly Expressed Genes in Infant C57BL/6 Mouse Hippocampus in Relation to Phenotypic Annotation Derived From Mutational Studies Click here for additional data file. Supplemental material, sj-xlsx-2-bbi-10.1177_11779322211062722 for The Vulnerability of the Developing Brain: Analysis of Highly Expressed Genes in Infant C57BL/6 Mouse Hippocampus in Relation to Phenotypic Annotation Derived From Mutational Studies by Angelica Lindlöf in Bioinformatics and Biology Insights

sj-xlsx-3-bbi-10.1177_11779322211062722 – Supplemental material for The Vulnerability of the Developing Brain: Analysis of Highly Expressed Genes in Infant C57BL/6 Mouse Hippocampus in Relation to Phenotypic Annotation Derived From Mutational Studies Click here for additional data file. Supplemental material, sj-xlsx-3-bbi-10.1177_11779322211062722 for The Vulnerability of the Developing Brain: Analysis of Highly Expressed Genes in Infant C57BL/6 Mouse Hippocampus in Relation to Phenotypic Annotation Derived From Mutational Studies by Angelica Lindlöf in Bioinformatics and Biology Insights

sj-xlsx-4-bbi-10.1177_11779322211062722 – Supplemental material for The Vulnerability of the Developing Brain: Analysis of Highly Expressed Genes in Infant C57BL/6 Mouse Hippocampus in Relation to Phenotypic Annotation Derived From Mutational Studies Click here for additional data file. Supplemental material, sj-xlsx-4-bbi-10.1177_11779322211062722 for The Vulnerability of the Developing Brain: Analysis of Highly Expressed Genes in Infant C57BL/6 Mouse Hippocampus in Relation to Phenotypic Annotation Derived From Mutational Studies by Angelica Lindlöf in Bioinformatics and Biology Insights

sj-xlsx-5-bbi-10.1177_11779322211062722 – Supplemental material for The Vulnerability of the Developing Brain: Analysis of Highly Expressed Genes in Infant C57BL/6 Mouse Hippocampus in Relation to Phenotypic Annotation Derived From Mutational Studies Click here for additional data file. Supplemental material, sj-xlsx-5-bbi-10.1177_11779322211062722 for The Vulnerability of the Developing Brain: Analysis of Highly Expressed Genes in Infant C57BL/6 Mouse Hippocampus in Relation to Phenotypic Annotation Derived From Mutational Studies by Angelica Lindlöf in Bioinformatics and Biology Insights
