# Supplementary figures and images for: The Vulnerability of the Developing Brain: Analysis of Highly Expressed Genes in Infant C57BL/6 Mouse Hippocampus in Relation to Phenotypic Annotation Derived From Mutational Studies
Source: Bioinform Biol Insights. 2022 Jan 5;16:11779322211062722. doi: 10.1177/11779322211062722 (PMC8743926; doi:10.1177/11779322211062722)

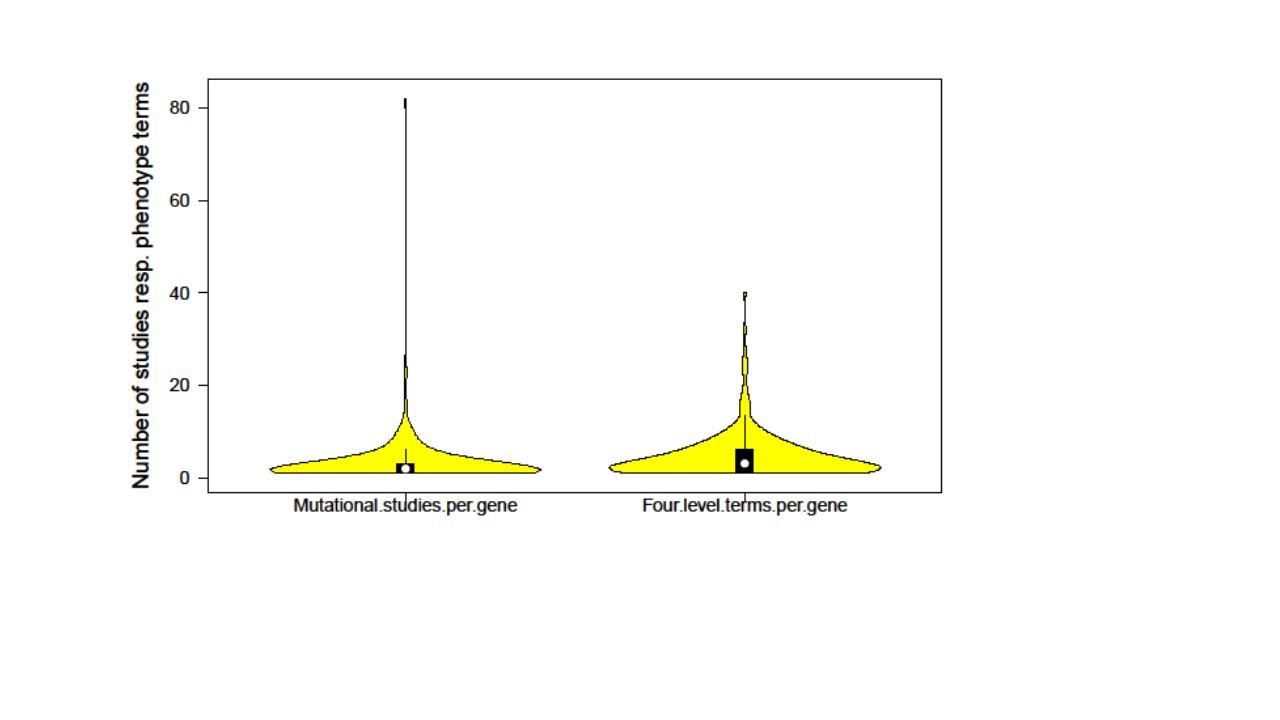

Supplement: sj-jpg-1-bbi-10.1177_11779322211062722 – Supplemental material for The Vulnerability of the Developing Brain: Analysis of Highly Expressed Genes in Infant C57BL/6 Mouse Hippocampus in Relation to Phenotypic Annotation Derived From Mutational Studies [file sj-jpg-1-bbi-10.1177_11779322211062722.jpg]
